# Bipolar Membrane Electrodialysis for Cleaner Production of Diprotic Malic Acid: Separation Mechanism and Performance Evaluation

**DOI:** 10.3390/membranes13020197

**Published:** 2023-02-05

**Authors:** Jinfeng He, Rong Zhou, Zhiguo Dong, Junying Yan, Xixi Ma, Wenlong Liu, Li Sun, Chuanrun Li, Haiyang Yan, Yaoming Wang, Tongwen Xu

**Affiliations:** 1 School of Pharmacy, Pharmaceutical Engineering Technology Research Center, Anhui University of Chinese Medicine, Hefei 230012, China; 2 Anhui Provincial Engineering Laboratory for Functional Membranes, Department of Applied Chemistry, School of Chemistry and Materials Science, University of Science and Technology of China, Hefei 230026, China; 3 Anhui Province Key Laboratory of Pharmaceutical Preparation Technology and Application, Hefei 230012, China

**Keywords:** bipolar membrane electrodialysis, diprotic malic acid, cleaner production, separation mechanism, performance evaluation

## Abstract

Bipolar membrane electrodialysis (BMED) is a promising process for the cleaner production of organic acid. In this study, the separation mechanism of BMED with different cell configurations, i.e., BP-A, BP-A-C, and BP-C (BP, bipolar membrane; A, anion exchange membrane; C, cation exchange membrane), to produce diprotic malic acid from sodium malate was compared in consideration of the conversion ratio, current efficiency and energy consumption. Additionally, the current density and feed concentration were investigated to optimize the BMED performance. Results indicate that the conversion ratio follows BP-C > BP-A-C > BP-A, the current efficiency follows BP-A-C > BP-C > BP-A, and the energy consumption follows BP-C < BP-A-C < BP-A. For the optimized BP-C configuration, the current density was optimized as 40 mA/cm^2^ in consideration of low total process cost; high feed concentration (0.5–1.0 mol/L) is more feasible to produce diprotic malic acid due to the high conversion ratio (73.4–76.2%), high current efficiency (88.6–90.7%), low energy consumption (0.66–0.71 kWh/kg) and low process cost (0.58–0.59 USD/kg). Moreover, a high concentration of by-product NaOH (1.3497 mol/L) can be directly recycled to the upstream process. Therefore, BMED is a cleaner, high-efficient, low energy consumption and environmentally friendly process to produce diprotic malic acid.

## 1. Introduction

Malic acid is a four-carbon dicarboxylic acid and an intermediate of the tricarboxylic acid cycle [1,2]. It has been used widely in food and pharmaceutical industries [2,3,4]. In the food industry, the malic acid is mainly used as acidulant and taste enhancer in various products including candies, low-caloric drinks and bakery products [2,3]. In the pharmaceutical industry, malic acid has some significant applications such as pH corrective, anti-oxidant and formulation of medicines (e.g., migraine drug almotriptan malate) [2,3]. Moreover, the malic acid can be used as a polishing or cleaning formulation compound in semiconductor fabrication, and as animal feed additives [2]. Furthermore, malic acid can be potentially used in the synthesis of bio-based polymers due to its dicarboxylic nature [2]. Based on the above applications, the global market of malic acid reaches to the range of 6 × 10^4^ to 2 × 10^5^ tons per year and is predicted to increase in the coming years [2].

Nowadays, enzymatic conversion from fumaric acid plays an important role in the industrial production of malic acid, which mainly includes fermentation, filtration, precipitation and acidification processes [3,4,5,6]. Herein, the acidification process such as the ion exchange [7] is required because the malic ions existed as their salts (e.g., sodium malate and potassium malate [4]) after the precipitation process. In the above separation processes, not only are a large amount of fresh chemicals consumed, but also waste or saline effluent is generated [3,5]. To overcome these downstream processing drawbacks, bipolar membrane electrodialysis (BMED) is a promising alternative due to its advantages of being chemical consumption free, high-efficient and environmentally friendly [8,9,10].

In the BMED process, water splitting will occur at the interface of the bipolar membrane when the applied current density exceeds the limiting current density of the bipolar membrane [11,12,13]. The dissociated H^+^ will react with organic anions to produce organic acid, while the dissociated OH^−^ will combine with metal ions, such as K^+^ and Na^+^ ions, to produce the corresponding by-product of a base that can be recycled to the upstream procedures for pH adjusting [14,15]. Typically, three kinds of configurations (i.e., BP-A, BP-A-C, BP-C configurations, BP-bipolar membrane, A-anion exchange membrane, C-cation exchange membrane) of BMED stack are feasible for the production of organic acids [15,16]. In recent years, BMED has been applied to the production of gluconic acid [14,15], citric acid [17], succinic acid [18], niacin [19], tartaric acid [20], L-10-camphorsulfonic acid [10], etc. Currently, however, there are only a limited number of reports about the cleaner production of malic acid by BMED [4,21,22,23]. For instance, Quoc et al. reported that the electro-acidification of cloudy apple juice containing malic acid was carried out by BMED with a BP-A configuration (no current efficiency and energy consumption data) [21,22]; Vera et al. reported a deacidification process of clarified tropical fruit juices, containing malic acid, by BMED with a BP-A configuration, in which the current efficiency and energy consumption are ~30% and ~0.13 kWh/kg, respectively, at a current density of 10 mA/cm^2^ [24]; Lameloise et al. reported the malic acid recovery from a beverage industry wastewater by BMED with a BP-C configuration, in which the conversion ratio, current efficiency and energy consumption of the malic acid were 93–97%, 87–97% and 1.15–1.27 kWh/kg, respectively, at a current density of ~50 mA/cm^2^ [4]; and Liu et al. reported a novel BMED process (BP-A-C configuration) integrated with a biochemical process for the malic acid production, in which the conversion ratio, current efficiency and energy consumption of the malic acid were 76.7% (calculated by 0.3 mol/L malate conversed into 0.23 mol/L malic acid), 70% and 0.34 kWh/kg at a low current density of ~1 mA/cm^2^ [23]. Nevertheless, the current investigations about malic acid production by BMED are not comprehensive enough, especially for the separation mechanism of different configurations at various operating parameters.

**Scheme 1:**
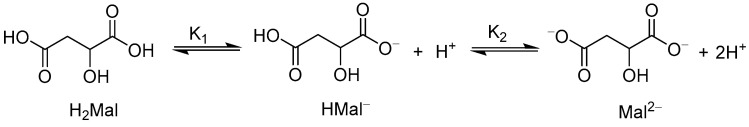
The dissociation reaction of malic acid.

**Figure 1:**
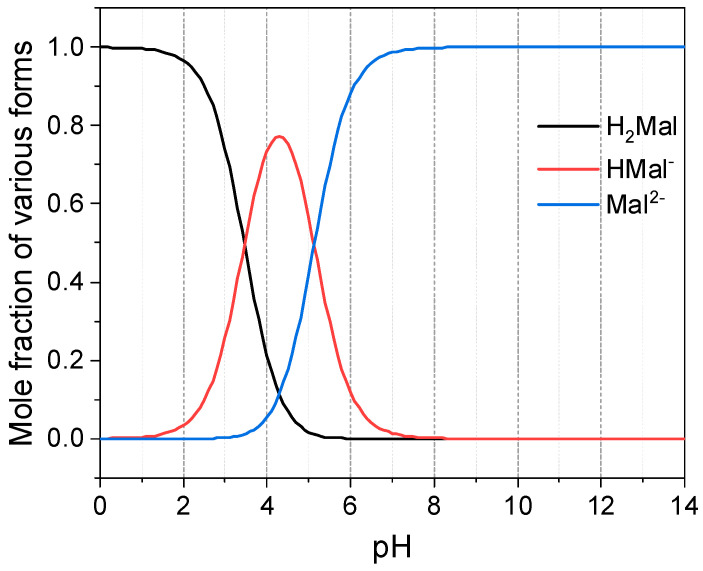
Mole fraction of various forms (H_2_Mal, HMal^−^ and Mal^2−^) at different surrounding pH.

As mentioned before, malic acid is a diprotic acid ($pK_{1}$ = 3.46, $pK_{2\;}$= 5.11 [3,4]), and its dissociation reaction is as follows (Scheme 1):

It means that the malate ions can be classified into three speciation of H_2_Mal, HMal^−^ and Mal^2−^ at different surrounding pH. According to the ionization equilibrium, the mole fraction of these three speciation are calculated by [25]:

(1) $$\delta_{H_{2}Mal}=\frac{1}{1+10^{pH-pK_{1}}+10^{2pH-pK_{1}-pK_{2}}}$$

(2) $$\delta_{HMal^{-}}=10^{pH-pK_{1}}\times \delta_{H_{2}Mal}$$

(3) $$\delta_{Mal^{2-}}=10^{2pH-pK_{1}-pK_{2}}\times \delta_{H_{2}Mal}$$

where $\delta_{H_{2}Mal}$, $\delta_{HMal^{-}}$ and $\delta_{Mal^{2-}}$ are the fraction ratios of H_2_Mal, HMal^−^ and Mal^2−^, respectively. The calculated values are shown in Figure 1, which indicates that the surrounding pH regulates the above speciation (i.e., H_2_Mal, HMal^−^ and Mal^2−^). We can find that the lower the solution pH, the higher the conversion ratio of malic acid. However, at a much lower pH, the concentration of H^+^ in the solution will be high, which would influence the ion migration in BMED with different stack configurations.

Hence, this study aims to investigate the separation mechanism of BMED with different cell configurations, i.e., BP-A, BP-A-C and BP-C configurations, in consideration of the conversion ratio, current efficiency and energy consumption. Additionally, operating parameters including current density and feed solution were investigated to optimize BMED performances. This work provides a better understanding of the BMED process for the high-efficient conversion of diprotic malic acid with low energy consumption.

## 2. Experimental Section

### 2.1. Materials

The bipolar membrane (BPM or BP) used in the experiments is BP-1E (Tokuyama Co., Japan). The cation exchange membrane (CEM or C) and anion exchange membrane (AEM or A) are CIS and AIS, respectively. Both membranes were provided by Shandong Tianwei Membrane Technology Co., Ltd., China. Their properties are listed in Table 1. Sodium malate (Na_2_Mal) was purchased from Aladdin Chemical Reagent Co., Ltd., China (98% purity). The chemical reagents, including Na_2_CO_3_, Na_2_SO_4_, HCl and NaOH, were purchased from Sinopharm Group Chemical Reagent Co., Ltd., China. All chemicals used were analytical grade. Deionized water was used.

### 2.2. BMED Setup

**Figure 2:**
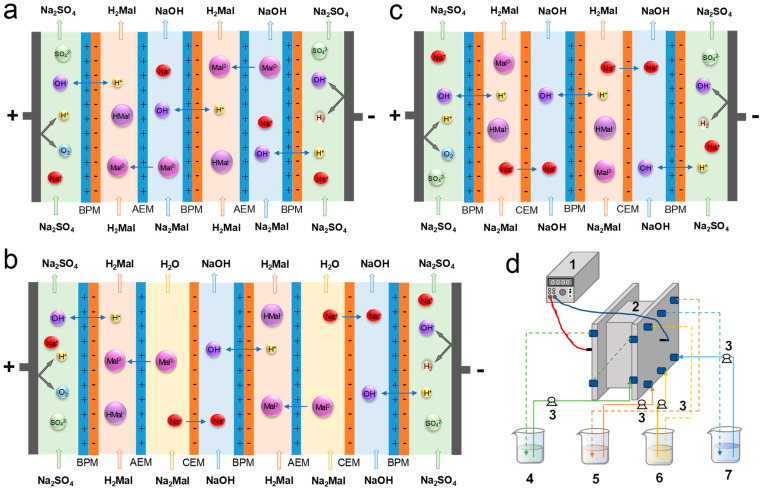
Schematic diagram of BP-A configuration (**a**), BP-A-C configuration (**b**), BP-C configuration (**c**), and BMED setup (**d**). Notes: 1, direct current supply; 2, BMED stack; 3, pump; 4, electrode rinse solution chamber; 5, acid solution chamber; 6, base solution chamber; 7, salt solution chamber (only for BP-A-C configuration).

As shown in Figure 2, three cell configurations (BP-A, BP-A-C and BP-C) were considered for the construction of a laboratory-scale BMED stack (CJED-1020, Hefei Chemjoy Polymer Materials Co., Ltd., China). Each configuration of membrane stack has two cell pairs, an anode compartment and a cathode compartment. In the membrane stack, the adjacent two membranes were separated by a spacer with a thickness of 0.75 mm. The effective area of each membrane was 189 cm^2^. Membranes used were arranged alternatively between the anode and cathode, which were made of titanium coated with ruthenium. These two electrodes were connected to a direct current power supply (HSPY-100-10, Beijing Hanshengpuyuan Science and Technology Co., Ltd., China). Specifically, in the BP-A configuration, the cell pair contained an acid compartment and a salt/base compartment; in the BP-A-C configuration, the cell pair comprised an acid compartment, a base compartment and a salt compartment; and in the BP-C compartment, the cell pair comprised of a salt/acid compartment and a base compartment. During the experiment, the solutions were pumped into the corresponding compartment at a flow rate of 4 cm/s. The electrode rinse solution was 400 mL 0.3 mol/L Na_2_SO_4_.

### 2.3. Experimental Procedure

In this study, the effects of the cell configuration, current density and feed concentration were investigated, respectively. Firstly, three kinds of configurations were applied to produce malic acid. In the case of the BP-A configuration, the feed solution (400 mL 0.5 mol/L Na_2_Mal) was pumped into the salt/base compartment, and 400 mL 0.05 mol/L H_2_Mal solution was pumped into the acid compartment as the initial solution. In the case of the BP-A-C configuration, the feed solution was pumped into the salt compartment, 400 mL 0.05 mol/L H_2_Mal solution was pumped into the acid compartment and 400 mL 0.1 mol/L NaOH solution was pumped into the base compartment. In the case of the BP-C configuration, the feed solution was pumped into the salt/acid compartment, and 400 mL 0.1 mol/L NaOH solution was pumped into the base compartment. Each batch operation was carried out at galvanostatic mode (20 mA/cm^2^). Secondly, various current densities (10, 20, 40 mA/cm^2^) were applied to the BMED stack with the optimized configuration. All the solutions pumped into the corresponding compartments were the same as the above mentioned. Lastly, various feed concentrations (0.25, 0.50, 1.00 mol/L Na_2_Mal) were investigated at the optimized configuration and current density. Meanwhile, during the experiment, the pH ($pH_{acid}$) and conductivity ($\sigma_{acid}$) of the acid solution were monitored by a pH meter (ST5000, OHAUS Instruments, USA) and a conductivity meter (DDBJ-350F, Shanghai INESA & Scientific Instrument Co., Ltd., China), respectively. The total produced H^+^ in the forms of H^+^, HMal^−^ and H_2_Mal was titrated by NaOH standard solution with the phenolphthalein as an indicator. In all cases, the produced NaOH was titrated by HCl standard solution with methyl orange as an indicator.

### 2.4. Data Analysis

The conversion ratio of H_2_Mal from Na_2_Mal was defined as:

(4) $$CR=\frac{C_{H^{+},total}\times V_{acid,\;t}}{C_{Na_{2}Mal,0}\times V_{salt,\;0}}\times 100\%$$

where $C_{H^{+},total}$ is the concentration of the total produced H^+^ in the acid compartment at time *t*, $C_{Na_{2}Mal,0}$ is the concentration of Na_2_Mal in the salt compartment at time 0, $V_{acid,\;t}$ is the volume of the acid solution at time *t*, and $V_{salt,\;0}$ is the volume of the salt solution at time 0.

The flux of NaOH produced ($J_{NaOH}$, mmol/(m^2^ s)) is calculated as:

(5) $$J_{NaOH}=\frac{C_{NaOH,t}\times V_{base,\;t}-C_{NaOH,0}\times V_{base,\;0}}{N\times A\times t}\times 100\%$$

where $C_{NaOH,t}$ and $C_{NaOH,0}$ are the concentration of NaOH in the base compartment at time *t* and 0, respectively, $V_{base,\;t}$ and $V_{base,\;0}$ are the volumes of base solution at time *t* and 0, respectively, *N* is the number of cell pairs (*N* = 2), *A* is the effective area of single membrane (189 cm^2^), and *t* is the running time of the experiment.

The current efficiency (CE, %) is defined as the ratio between the number of ions in equivalent molar weight transported through the membrane and the quantity of electric charge consumed:

(6) $$CE=\frac{\Delta n\times F}{N\times I\times t}\times 100\%$$

where Δn is the number of equivalents transferred through membrane, *F* is the Faraday constant (96485 C/equiv.) and *I* (A) is the current. It should be noted that Δn refers to Mal^2−^ and Na^+^ for BP-A and BP-C configurations, respectively, and refers to Mal^2−^ or Na^+^ for the BP-A-C configuration.

The energy consumption of the production of H_2_Mal (EC, kWh/kg) in the BMED process, excluding anode and cathode compartments, was calculated by [10]:

(7) $$EC=\frac{\int_{0}^{t}N\times V_{cell}\times Idt}{\frac{C_{H^{+},total}}{2}\times V_{acid,\;t}\times M}=\frac{2\int_{0}^{t}N\times V_{cell}\times Idt}{C_{H^{+},total}\times V_{acid,\;t}\times M}$$

where *M* is the molar weight of H_2_Mal (134 g/mol), and $V_{cell}$ (V) is the voltage drop across one cell pair. Here, we defined a half of $C_{H^{+},total}$ as the concentration of the produced H_2_Mal in the acid compartment ignoring the ionization equilibrium. The $V_{cell}$ was determined by [26]:

(8) $$V_{cell}=\frac{V_{stack}-V_{EC}}{N}$$

where $V_{stack}$ (V) is the voltage drop across the membrane stack, and $V_{EC}$ (V) is the voltage drop between the anode and cathode compartments, which was measured and shown in Figure S1 in Supplementary Materials.

## 3. Results and Discussion

### 3.1. Effect of the Cell Configuration

#### 3.1.1. Separation Mechanism for Different Cell Configurations

In the BMED process, a different cell configuration means a different separation mechanism, as shown in Figure 2. Here, three different cell configurations, i.e., BP-A, BP-A-C and BP-C, were applied to produce diprotic malic acid. The feed solution was 0.5 mol/L Na_2_Mal. The BMED was operated at a current density of 20 mA/cm^2^.

**Figure 3:**
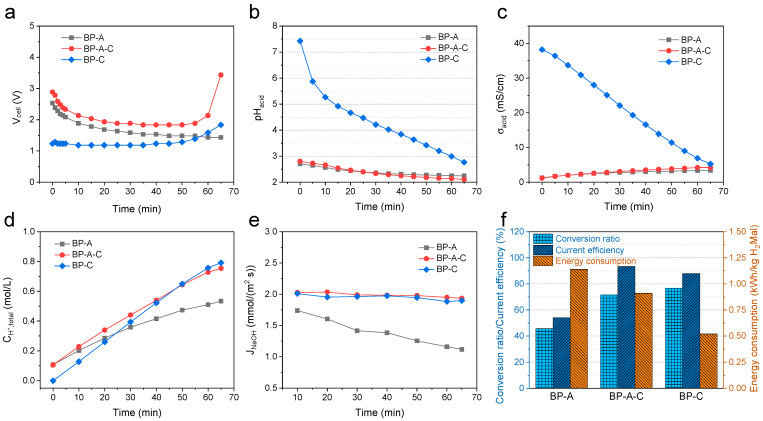
Effect of the cell configuration of BMED performances: (**a**) $V_{cell}$, (**b**) $pH_{acid}$, (**c**) $\sigma_{acid}$, (**d**) $C_{H^{+},total}$, (**e**) $J_{NaOH}$, and (**f**) conversion ratio, current efficiency and energy consumption. Notes: feed concentration, 0.5 mol/L; current density, 20 mA/cm^2^.

In the case of BP-A configuration, the feed solution was pumped into the salt/base compartment, then, under the driving force of the direct current, the Mal^2−^ ions migrated through the AEM to the adjacent acid compartment and reacted with the produced H^+^ ions (water dissociation inside the BPM, Figure 2) to produce H_2_Mal, resulting in a decrease in $pH_{acid}$ and an increase in $C_{H^{+},total}$ (Figure 3b,d). Meanwhile, the Na^+^ ions combined with the produced OH^−^ ions (water dissociation inside the BPM, Figure 2) to produce the by-product NaOH (Figure S2 and Figure 3e). As OH^−^ ions have a smaller ionic size (bare radius of 1.76 Å) compared with Mal^2−^ ions (bare radius of 3.01 Å) shown in Table 2, these ions in the base compartment would compete with Mal^2−^ ions to migrate through the AEM to the acid compartment, decreasing the growth of $C_{H^{+},total}$ (Figure 3d) and $J_{NaOH}$ (Figure 3e). Nevertheless, both the increasing $\sigma_{acid}$ of the acid solution (Figure 3c) and $C_{NaOH}$ of the base solution (Figure S2 in Supplementary Materials) reduces the stack resistance, resulting in the decreasing $V_{cell}$ shown in Figure 3a.

For the BP-A-C configuration, the feed solution was pumped into the salt compartment, then, like the BP-A configuration, the Mal^2−^ ions migrated through the AEM to the adjacent acid compartment and reacted with the produced H^+^ ions to produce H_2_Mal. But unlike the BP-A configuration, the Na^+^ ions in the feed migrated through the CEM to the adjacent base compartment and combined with the produced OH^−^ ions to produce NaOH. Due to this separation mechanism, a lower $pH_{acid}$ and a higher $C_{H^{+},total}$ can be reached for the BP-A-C configuration compared with the BP-A configuration (Figure 3b,d). Additionally, the $J_{NaOH}$ for the BP-A-C configuration is higher than that for BP-A configuration (Figure 3e). What is more, the salt conductivity ($\sigma_{salt}$) decreases gradually as a function of time, as shown in Figure S3 in Supplementary Materials. This means that the resistance of the salt solution increases gradually along the experiment, resulting in a rapid increase in $V_{cell}$ at the end of the experiment (Figure 3a).

For the last configuration, i.e., the BP-C configuration, the feed solution was pumped into the salt/acid compartment; unlike the BP-A and BP-A-C configurations, the Mal^2−^ ions retained in the salt/acid compartment. Meanwhile, the Na^+^ ions in the feed solution migrated through the CEM to the adjacent base compartment. In the salt/acid compartment, the Mal^2−^ ions reacted with the produced H^+^ ions, resulting in the transformation of ionization equilibrium from Mal^2−^ ions to HMal^−^ ions firstly (Figure 1). As the $pH_{acid}$ decreases, the ionization equilibrium would transform from HMal^−^ ions to H_2_Mal. In this process, the produced H^+^ ions were almost reacted with Mal^2−^ ions, because the concentration of free H^+^ ions in the acid compartment ($C_{H^{+}}$) is as low as < 0.0017 mol/L (see Figure S4 20 mA/cm^2^ in Supplementary Materials). Accordingly, there is a quite weaker competition of H^+^ ions with Na^+^ to migrate to the base compartment, resulting in a relatively high $J_{NaOH}$ that is approximately equal to that of the BP-A-C configuration (Figure 3e). However, $C_{H^{+},total}$ of the BP-C configuration increases more rapidly than that of the BP-A-C configuration, which can be attributed to the reason that the migration of Na^+^ ions through the CEM is easier than the migration of Mal^2−^ ions through the AEM. This is because Na^+^ is a monovalent ion and has a small ionic size (bara radius of 0.95 Å) and high diffusion coefficient (1.33 × 10^−9^ m^2^/s), as shown in Table 2, while Mal^2−^ is a divalent ion and has a bigger ion size (bare radius of 3.01 Å) and lower diffusion coefficient (9.46 × 10^−10^ m^2^/s) compared with Na^+^ ions. In addition, the free volume hole radius of the commercial membranes is generally in the range of 2.2–2.8 Å [10], smaller than that of the bare radius of Mal^2−^ ions. What is more, the decreasing $\sigma_{acid}$ (Figure 3c) increases the resistance of the salt/acid solution, resulting in a gradual increase in $V_{cell}$ at the end of the experiment (Figure 3a).

#### 3.1.2. Conversion Ratio, Current Efficiency and Energy Consumption

The BMED performances including the conversion ratio, current efficiency and energy consumption for three different configurations were evaluated, as shown in Figure 3f. We can see the conversion ratio follows the order: BP-C (76.7%) > BP-A-C (71.6%) > BP-A (45.7%), which is consistent with the $C_{H^{+},total}$ shown in Figure 3d. The low conversion ratio for the BP-A configuration is caused by the migration of OH^−^ ions competing with Mal^2−^ ions to the acid compartment. The higher conversion ratio for the BP-C configuration compared with the BP-A-C configuration is due to the special separation mechanism (i.e., the migration of Na^+^ ions through the CEM is easier than the migration of Mal^2−^ ions through the AEM) mentioned before. Moreover, the current efficiency follows the order: BP-A-C (93.3%) > BP-C (88.0%) > BP-A (54.1%), which is consistent with $J_{NaOH}$. The reason for the lowest current efficiency for the BP-A configuration is the same as that described for the conversion ratio. In the BP-C configuration, the migration of H^+^ ions from the acid compartment through the CEM to the base compartment decreases the current efficiency. However, this phenomenon cannot occur in the case of the BP-A-C configuration; thus, the current efficiency of which is higher than that of the BP-C configuration. At last, the energy consumption follows the order of BP-C (0.52 kWh/kg) < BP-A-C (0.91 kWh/kg) < BP-A (1.14 kWh/kg). The lowest energy consumption for the BP-C configuration is due to the highest conversion ratio and lowest $V_{cell}$ (Figure 3a) according to Equations (4) and (7). The highest energy consumption for the BP-A configuration is mainly caused by the lowest conversion ratio, as mentioned above, though the $V_{cell}$ is lower than that of the BP-A-C configuration. Overall, the cell configuration was optimized as the BP-C configuration due to a high conversion ratio (76.7%), relatively high current efficiency (88.0%) and low energy consumption (0.52 kWh/kg).

### 3.2. Effect of the Current Density

As mentioned above, in the BP-C configuration, although the $C_{H^{+}}$ of the acid compartment is much lower, the migration of free H^+^ ions from the acid compartment through the CEM to the base compartment can slightly reduce the $J_{NaOH}$ due to the acid-base neutralization, resulting in a decrease in current efficiency. Therefore, various current densities (10, 20 and 40 mA/cm^2^) were applied to the BMED stack with the BP-C configuration to investigate the separation mechanism, especially for the migration of free H^+^ ions from the acid compartment to the base compartment. The feed solution was 0.5 mol/L Na_2_Mal. Each batch operation consumes the same number of coulombs (*It*).

**Figure 4:**
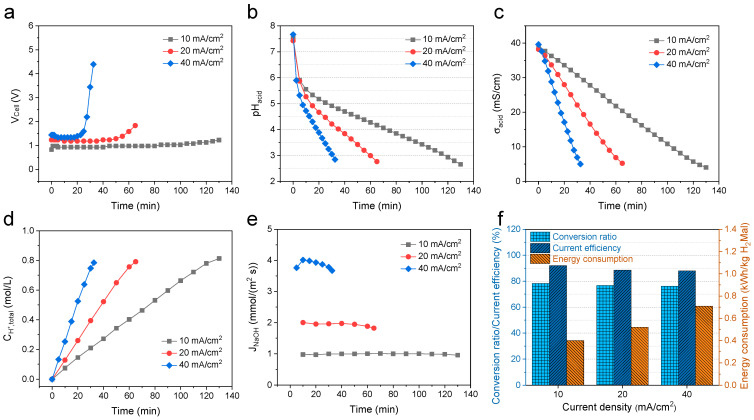
Effect of the current density of BMED performances: (**a**) $V_{cell}$, (**b**) $pH_{acid}$, (**c**) $\sigma_{acid}$, (**d**) $C_{H^{+},total}$, (**e**) $J_{NaOH}$, and (**f**) conversion ratio, current efficiency and energy consumption. Notes: BP-C configuration; feed concentration, 0.5 mol/L.

Figure 4b shows that the $pH_{acid}$ decreases as the time prolongs due to the continuous dissociation of H^+^ by the BPM. Consistently, the $C_{H^{+},total}$ increases gradually as a function of time (Figure 4d). Interestingly, as the current density increases from 10 to 40 mA/cm^2^, the final $pH_{acid}$ increases from 2.66 to 2.84, and the final $C_{H^{+}}$ decreases from 0.0022 to 0.0014 mol/L (Figure S4 in Supplementary Materials). Similarly, the final $C_{H^{+},total}$ decreases from 0.8135 to 0.7852 mol/L with the increasing current density. The reason can be ascribed to the migration of free H^+^ ions from the acid compartment to the base compartment, as mentioned before. Meanwhile, the higher the current density, the more the free H^+^ ions migrated through the CEM from the acid compartment to the base compartment. Therefore, the conversion ratio has a slight decrease (from 78.4% to 76.2%) as the current density increases, as shown in Figure 4f.

Figure S5 (in Supplementary Materials) shows the $C_{NaOH}$ increases gradually as a function of time, and the running time of the batch experiment can be shortened at a higher current density. Specifically, the $J_{NaOH}$ exhibits a decreasing trend as a function of time, as shown in Figure 4e, which should be ascribed to the migration of free H^+^ ions. More importantly, the higher the current density, the more the decrease in $J_{NaOH}$. Accordingly, the current efficiency decreases a lot from 92.0% to 88.6% as the current density increases from 10 to 20 mA/cm^2^, and then decreases a little to 88.0% as the current density increases to 40 mA/cm^2^ (Figure 4f).

Figure 4c shows that the $\sigma_{acid}$ decreases as a function of time, because the Mal^2−^ ions in the acid compartment reacted with the produced H^+^ ions, resulting in the transformation of ionization equilibrium from Mal^2−^ ions to HMal^−^ ions and H_2_Mal. The reduction of $\sigma_{acid}$ increases the acid solution resistance, enhancing the $V_{cell}$ at the end of the experiment, as shown in Figure 4a. Additionally, Figure 4a shows that the $V_{cell}$ increases with the increasing current density, which is consistent with Ohm’s law [14]. Moreover, the energy consumption increases gradually from 0.40 to 0.71 kWh/kg, as shown in Figure 4f, which is lower than the reported values of 1.15–1.27 kWh/kg at a current density of ~50 mA/cm^2^ [4]. Nevertheless, the high current density can obviously shorten the running time. It means that less investment is required for the high current density. Table 3 shows the calculation of the total fixed cost of the BMED apparatus, which is 168.32 USD/year. Table 4 shows the estimation of the total process cost for various current density. The treatment capacity increases from 81.3 to 323.4 kg/year as the current density increases from 10 to 40 mA/cm^2^, because shorter running time was needed at higher current density as mentioned before. Accordingly, the total fixed cost decreases dramatically as the current density increases. The total process cost is the sum of the energy consumption and total fixed cost. From the Table 4, we can see that the total fixed cost is the predominant cost of the total process cost, and the total process cost decreases from 2.10 USD/kg to 0.58 USD/kg dramatically. In overall, the current density was optimized as 40 mA/cm^2^ in consideration of low total process cost though the conversion ratio and current efficiency slightly lower than that of a lower current density.

### 3.3. Effect of the Feed Concentration

Based on the above, feed concentration plays an important role on ion migration as well as solution resistance, which affects the BMED performances. Hence, the effect of feed concentration (0.25, 0.5, 1.0 mol/L) was investigated. The current density applied was 40 mA/cm^2^.

**Figure 5:**
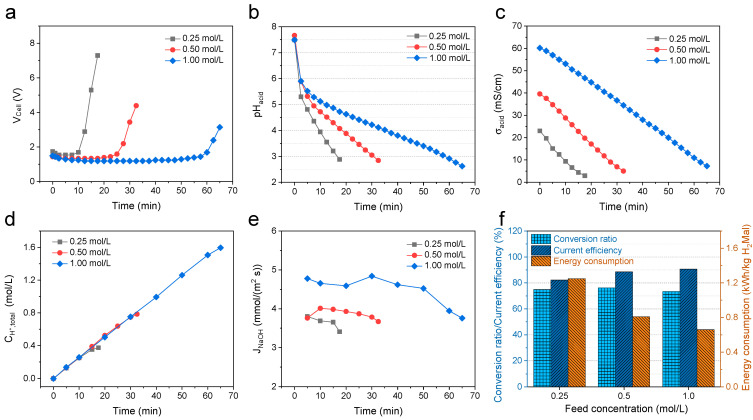
Effect of the feed concentration of BMED performances: (**a**) $V_{cell}$, (**b**) $pH_{acid}$, (**c**) $\sigma_{acid}$, (**d**) $C_{H^{+},total}$, (**e**) $J_{NaOH}$, and (**f**) conversion ratio, current efficiency and energy consumption. Notes: BP-C configuration; current density, 40 mA/cm^2^.

Figure 5b,d shows that the $pH_{acid}$ and $C_{H^{+},total}$ decreases and increases, respectively, as a function of time. It is interesting that, in Figure 5b, the final $pH_{acid}$ decreases from 2.88 to 2.62 as the feed concentration increases. Consistently, the final $C_{H^{+}}$ increases from 0.0013 to 0.0024 mol/L with the increasing feed concentration. This is because the concentrations of H_2_Mal and HMal^−^ in the acid solution in the case of 1.0 mol/L feed concentration are higher than that in the other two cases. Then, the $C_{H^{+}}$ in the case of 1.0 mol/L feed concentration is higher than that in the other two cases according to the electroneutrality (Figure S6 in Supplementary Materials). According to the $C_{H^{+},total}$, the conversion ratio was calculated, as shown in Figure 5f. The conversion ratios are in the range of 73.4–76.2%, which can be increased by extending the running time.

Figure 5e shows that the $J_{NaOH}$ decreases gradually as a function of time due to the migration of free H^+^ ions mentioned before. Additionally, the $J_{NaOH}$ increases with the increasing feed concentration. The reason can be attributed to two aspects, as follows. On the one hand, a high concentration gradient of Na^+^ ions between the acid compartment and base compartment, at the former stage of the experiment, can enhance the migration of Na^+^ ions through the CEM according to the Nernst–Planck equation [34]. On the other hand, the high concentration of H_2_Mal and HMal^−^ in the acid compartment needs more H^+^ ions to maintain the electroneutrality of the acid solution, which prevents the migration of H^+^ ions through the CEM from the acid compartment to the base compartment to neutralize the produced NaOH. Moreover, the $C_{NaOH}$ increases from 0.4258 to 1.3497 mol/L with the increasing feed concentration, as shown in Figure S7 in Supplementary Materials. The by-product NaOH solution with the high concentration can be directly recycled to the upstream process [35]. Based on the $J_{NaOH}$, the current efficiencies were calculated and increased gradually from 82.3% to 90.7% as the feed concentration increased from 0.25 to 1.0 mol/L (Figure 5f).

Figure 5c shows that the overall $\sigma_{acid}$ decreases as the feed concentration increases, resulting in the increasing resistance of the acid solution and the increasing $V_{cell}$, as shown in Figure 5a. Then, the energy consumptions were calculated, which decreases from 1.25 to 0.66 kWh/kg with the increasing feed concentration shown in Figure 5f. In addition, the total process costs were estimated and listed in Table 5. As for the treatment capacity, it has the lowest value (294.3 kg/year) at the feed concentration of 0.25 mol/L since the $C_{H^{+},total}$ has a slight increase, rather than linear increase, at the end of experiment, as shown in Figure 5d. The treatment capacity increases to 323.4 kg/year as the feed concentration increases to 0.5 mol/L. Afterwards, the treatment capacity has a slight decrease as the feed concentration further increases to 1.0 mol/L (312 kg/year). The reason can be ascribed to the intensive migration of free H^+^ ions from the acid compartment to the base compartment because the final $C_{H^{+}}$ at the feed concentration of 1.0 mol/L is about 1.6 times of that at the feed concentration of 0.5 mol/L (Figure S6 in Supplementary Materials). Accordingly, the highest total fixed cost (0.57 USD/kg) is required at the feed concentration of 0.25 mol/L, and the lowest total fixed cost (0.52 USD/kg) at the feed concentration of 0.5 mol/L. Consequently, a high total process cost is required at low feed concentration (i.e., 0.25 mol/L), while the total process costs for cases of 0.5 mol/L and 1.0 mol/L are similar (0.58–0.59 USD/kg). Hence, high feed concentration (0.5–1.0 mol/L) is more feasible for the BMED process with the BP-C configuration to produce diprotic malic acid due to a high conversion ratio (73.4–76.2%), high current efficiency (88.6–90.7%), low energy consumption (0.66–0.71 kWh/kg) and low process cost (0.58–0.59 USD/kg).

## 4. Conclusions

In this study, a cleaner BMED process was proposed to efficiently convert sodium malate to produce diprotic malic acid. Three different cell configurations, i.e., BP-A, BP-A-C and BP-C, were investigated in consideration of separation mechanism, conversion ratio, current efficiency and energy. Results indicate that the BP-C configuration is preferable since the special separation mechanism of the migration of Na^+^ ions through the CEM from the salt/acid compartment to the base compartment, rather than the migration of Mal^2−^ ions through the AEM from the salt compartment to the acid compartment. Specifically, the conversion ratio follows BP-C > BP-A-C > BP-A, the current efficiency follows BP-A-C > BP-C > BP-A and the energy consumption follows BP-C < BP-A-C < BP-A. Additionally, various current densities (10–40 mA/cm^2^) and feed concentrations (0.25–1.0 mol/L) were investigated to optimize the BMED performance (BP-C configuration). Results indicate that the current density was optimized as 40 mA/cm^2^ in consideration of low total process cost and the high feed concentration (0.5–1.0 mol/L) is more feasible to produce malic acid due to the high conversion ratio (73.4–76.2%), high current efficiency (88.6–90.7%), low energy consumption (0.66–0.71 kWh/kg) and low process cost (0.58–0.59 USD/kg). Moreover, the by-product (NaOH) with a high concentration (1.3497 mol/L) can be directly recycled to the upstream process. Therefore, BMED is a cleaner, high-efficient, low energy consumption and environmentally friendly process to produce diprotic malic acid. Further studies are needed to enhance the conversion ratio and scale-up application for the real solution containing impurities (e.g., fumaric acid and fumarate).

## Figures and Tables

**Table 1:** Properties of BP-1E, CIS and AIS membranes.

Membrane Name	Thickness (μm)	IEC (meq/g)	Water Uptake (%)	Burst Strength (MPa)	Area Resistance (Ω·cm^2^)	Transport Number (%)
^a^ CIS	70	0.90–1.10	20–30	≥0.22	≤4.0	≥95
^a^ AIS	70	0.90–1.10	20–30	≥0.17	≤4.0	≥98
Membrane	Thickness (μm)	Water splitting voltage (V)	Water splitting efficiency	Burst strength (MPa)		
^b^ BP-1E	220	1.2	≥0.98	≥0.40		

^a^ The data obtained from the ShanDong TianWei membrane technology Co., Ltd., China. ^b^ The data obtained from the website of http://www.astom-corp.jp/en/product/05.html#01 (accessed on 1 January 2023).

**Table 2:** Intrinsic properties of Mal^2−^, HMal^−^, Na^+^, H^+^ and OH^−^.

Molecular Formula	Mal^2−^ (C_4_H_4_O_5_^2−^)	HMal^−^ (C_4_H_5_O_5_^−^)	Na^+^	H^+^	OH^−^
Molecular weight (g/mol)	132.09	133.09	22.99	1.00	17.00
Van der Waals volume (Å^3^)	^a^ 113.79	^a^ 115.11	-	-	-
Bare radius (Å)	^b^ 3.01	^b^ 3.02	^c^ 0.95	^c^ 0.28	^c^ 1.76
Hydrated radius (Å)	-	-	^c^ 3.58	^c^ 2.82	^c^ 3.00
Diffusion coefficient (m^2^/s)	^d^ 9.46 × 10^−10^	^d^ 9.40 × 10^−10^	^e^ 1.33 × 10^−9^	^f^ 9.31 × 10^−9^	^f^ 5.27 × 10^−9^

^a^ The van der Waals volume was calculated by a reported method [27]. ^b^ the radius was calculated by ${\left(\frac{3V}{4\pi }\right)}^{\frac{1}{3}}$, *V* is Van der Waals volume. ^c^ the data were obtained from [28]. ^d^ the diffusion coefficient was calculated by a method reported in [27]. ^e^ the data were obtained from [29]. ^f^ the data were obtained from [30].

**Table 3:** Estimation of the total fixed cost of BMED with BP-C configuration.

Parameters		Remarks
Membrane area of CIS (cm^2^)	594	11 × 27 × 2
Membrane area of BPM (cm^2^)	594	11 × 27 × 2
^a^ Membrane price of CIS (USD/m^2^)	122	
^b^ Membrane price of BPM (USD/m^2^)	1350	
Membrane cost (USD)	87.44	
Membrane lifetime and amortization of the peripheral equipment (year)	3	
Stack cost (USD)	131.16	×1.5 membrane cost
Peripheral equipment cost (USD)	196.73	×1.5 stack cost
Total investment cost (USD)	327.89	Stack cost + peripheral equipment cost
Amortization (USD/year)	109.30	3 years
Interest (USD/year)	26.23	Interest rate, 8%
Maintenance (USD/year)	32.79	10% of total investment cost
Total fixed cost (USD/year)	168.32	

^a^ The data obtained from the reference [31]; ^b^ The data obtained from the reference [32].

**Table 4:** Estimation of the total process cost for various current density.

Current Density (mA/cm^2^)	10	20	40
Energy consumption (kWh/kg)	0.40	0.52	0.71
Treatment capacity (kg/year)	81.3	166.6	323.4
Total fixed cost (USD/year)	168.32	168.32	168.32
Total fixed cost (USD/kg)	2.07	1.01	0.52
^a^ Total process cost (USD/kg)	2.10	1.05	0.58

^a^ The electricity price is 0.0825 USD/kWh [33].

**Table 5:** Estimation of the total process cost for various feed concentrations.

Feed Concentration (mol/L)	0.25	0.50	1.00
Energy consumption (kWh/kg)	1.25	0.71	0.66
Treatment capacity (kg/year)	294.3	323.4	312.0
Total fixed cost (USD/year)	168.32	168.32	168.32
Total fixed cost (USD/kg)	0.57	0.52	0.54
^a^ Total process cost (USD/kg)	0.67	0.58	0.59

^a^ The electricity price is 0.0825 USD/kWh [33].

## Data Availability

The data presented in this study are available on request from the corresponding author.

## Supplementary Materials

The following supporting information can be downloaded at: https://www.mdpi.com/article/10.3390/membranes13020197/s1, Figure S1: Voltage drops between the anode and cathode compartments ($V_{\mathrm{EC}}$) as a function of current (I); Figure S2: The concentration of NaOH in base compartment for different configurations as a function of time. Notes: current density, 20 mA/cm^2^; feed concentration, 0.5 mol/L; Figure S3: The conductivity of salt solution ($\sigma_{salt}$) using BP-A-C configuration as a function of time. Notes: current density, 20 mA/cm^2^; feed concentration, 0.5 mol/L; Figure S4: The concentration of H^+^ in acid compartment at various current densities as a function of time; Figure S5: The concentration of NaOH in base compartment at various current densities as a function of time; Figure S6: The concentration of H^+^ in acid compartment at various feed concentrations as a function of time; Figure S7: The concentration of NaOH in base compartment at various feed concentrations as a function of time.
