# Supplementary material for: Bipolar Membrane Electrodialysis for Cleaner Production of Diprotic Malic Acid: Separation Mechanism and Performance Evaluation
Source: Membranes (Basel). 2023 Feb 5;13(2):197. doi: 10.3390/membranes13020197 (PMC9961052; doi:10.3390/membranes13020197)
Supplement: Supplementary file 1 [file membranes-13-00197-s001.zip › membranes-2175039-SI.pdf]

membranes

Supplementary Materials

Bipolar Membrane Electrodialysis for Cleaner Production of Diprotic Malic Acid: Separation Mechanism and Performance Evaluation

Jinfeng He [1,†], Rong Zhou [1,†], Zhiguo Dong [1], Junying Yan [2], Xixi Ma [1], Wenlong Liu [1], Li Sun [1,3], Chuanrun Li [1,3], Haiyang Yan [1,3,*], Yaoming Wang [2] and Tongwen Xu [2,*]

1 School of Pharmacy, Pharmaceutical Engineering Technology Research Center, Anhui University of Chinese Medicine, Hefei 230012, China
2 Anhui Provincial Engineering Laboratory for Functional Membranes, Department of Applied Chemistry, School of Chemistry and Materials Science, University of Science and Technology of China, Hefei 230026, China
3 Anhui Province Key Laboratory of Pharmaceutical Preparation Technology and Application, Hefei 230012, China
* Correspondence: oceanyan@ustc.edu.cn (H.Y.); twxu@ustc.edu.cn (T.X.)
† These authors contributed equally to this work.

Citation: He, J.; Zhou, R.; Dong, Z.; Yan, J.; Ma, X.; Liu, W.; Sun, L.; Li, C.; Yan, H.; Wang, Y.; et al. Bipolar Membrane Electrodialysis for Cleaner Production of Diprotic Malic Acid: Separation Mechanism and Performance Evaluation. *Membranes* **2023**, *13*, 197. https://doi.org/10.3390/membranes13020197

Academic Editor: Seunghyeon Moon

Received: 5 January 2023
Revised: 19 January 2023
Accepted: 2 February 2023
Published: 5 February 2023

Copyright: © 2023 by the authors. Licensee MDPI, Basel, Switzerland. This article is an open access article distributed under the terms and conditions of the Creative Commons Attribution (CC BY) license (https://creativecommons.org/licenses/by/4.0/).

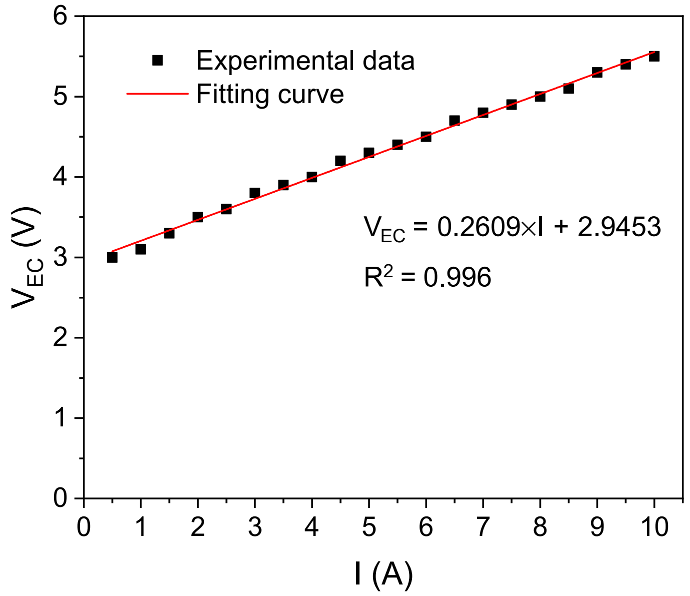

Figure S1. Voltage drops between the anode and cathode compartments (V_{EC}) as a function of current (I). Notes: The V_{EC} was measured in a stack that assembled only one piece of bipolar membrane, and the electrode rinse solution was 0.3 mol/L Na_2SO_4.

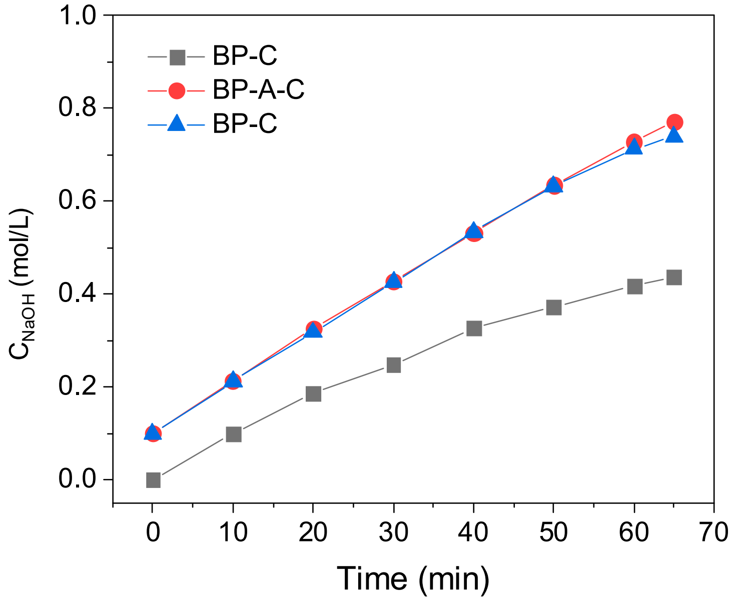

Figure S2. The concentration of NaOH in base compartment for different configurations as a function of time. Notes: current density, 20 mA/cm^2; feed concentration, 0.5 mol/L.

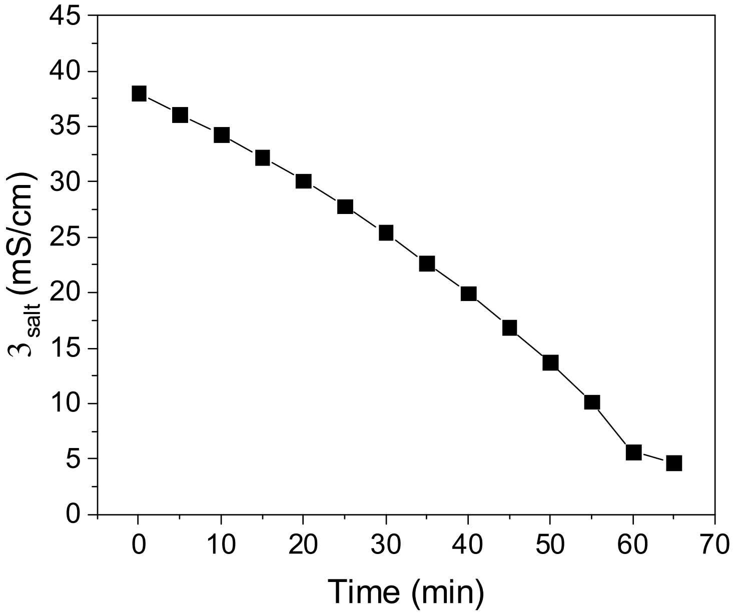

Figure S3. The conductivity of salt solution (σ_{salt}) using BP-A-C configuration as a function of time. Notes: current density, 20 mA/cm^2; feed concentration, 0.5 mol/L.

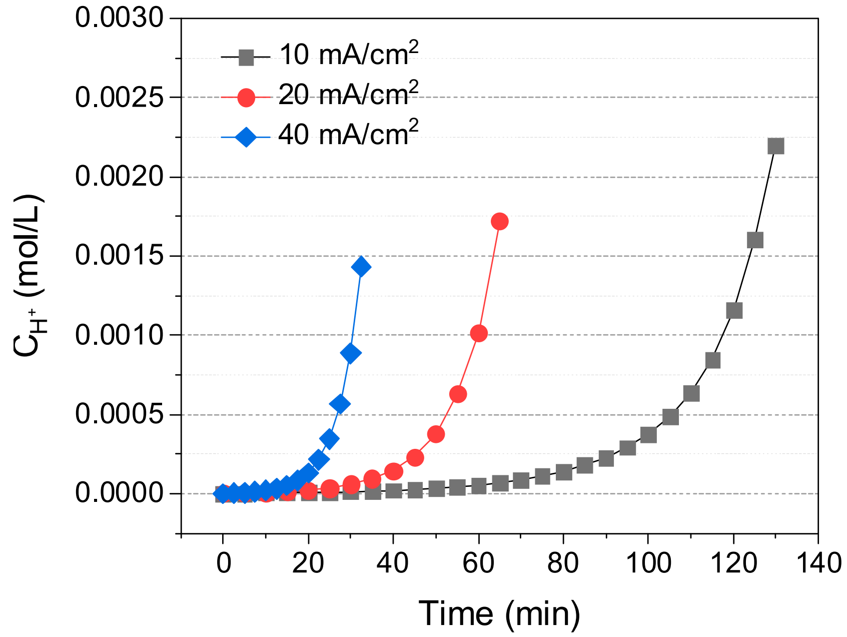

Figure S4. The concentration of H⁺ in acid compartment at various current densities as a function of time. Notes: the concentration of H⁺ was calculated from the solution pH; BP-C configuration; feed concentration, 0.5 mol/L.

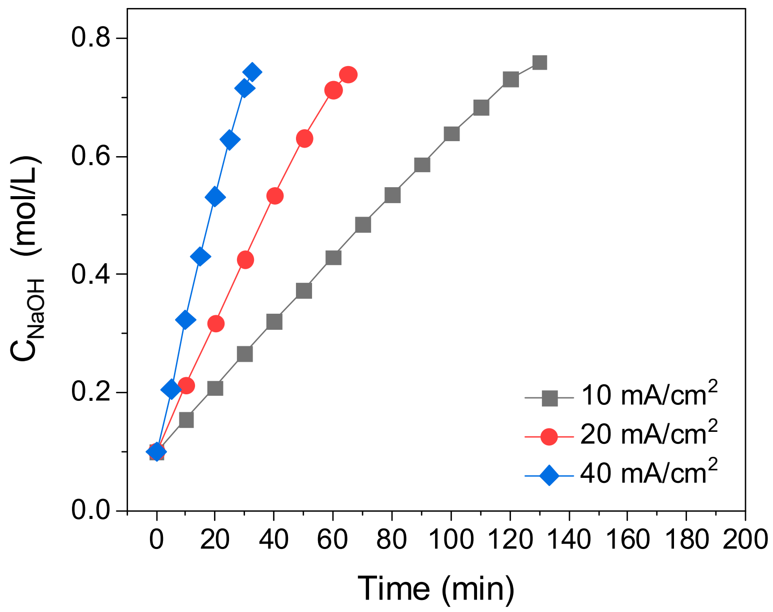

Figure S5. The concentration of NaOH in base compartment at various current densities as a function of time. Notes: BP-C configuration; feed concentration, 0.5 mol/L.

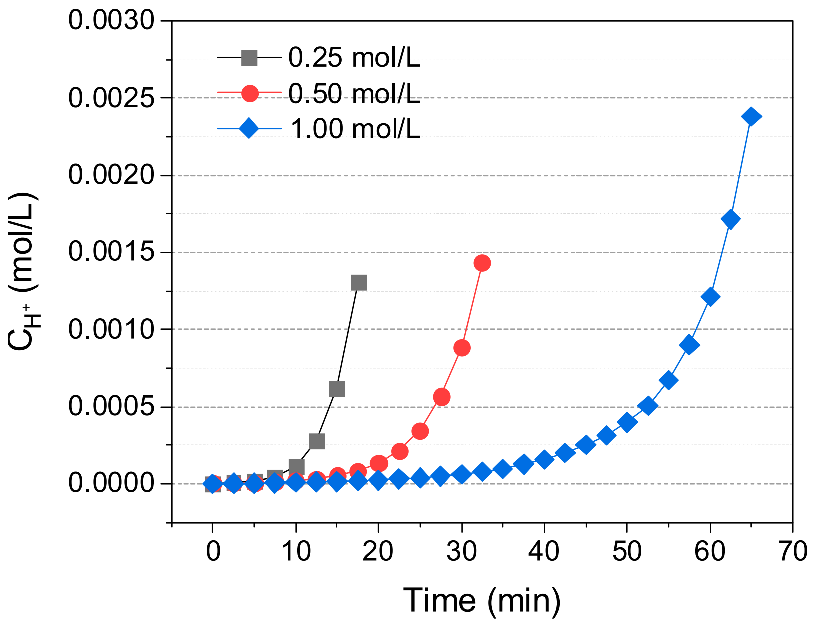

Figure S6. The concentration of H^+ in acid compartment at various feed concentrations as a function of time. Notes: the concentration of H^+ was calculated from the solution pH; BP-C configuration; current density, 40 mA/cm².

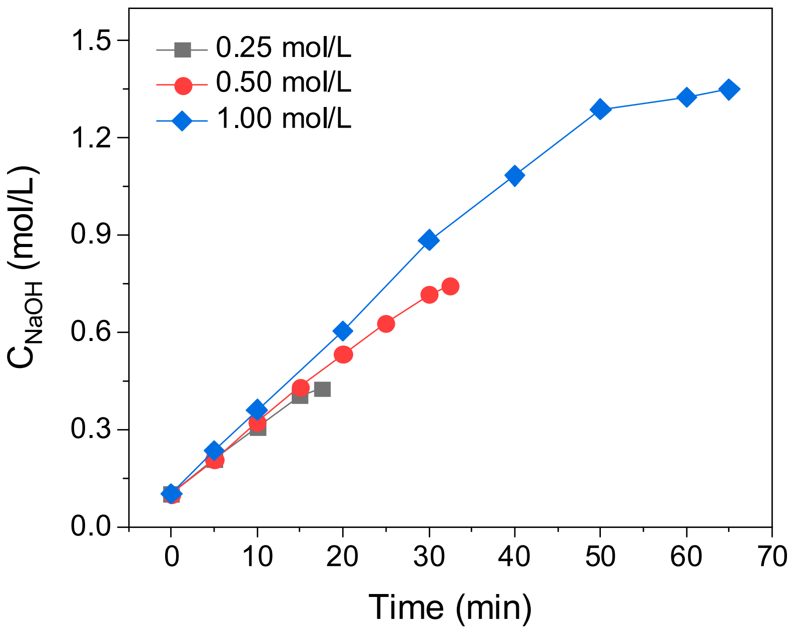

Figure S7. The concentration of NaOH in base compartment at various feed concentrations as a function of time. Notes: BP-C configuration; current density, 40 mA/cm².
